# Supplementary material for: A thousand-genome panel retraces the global spread and adaptation of a major fungal crop pathogen
Source: Nat Commun. 2023 Feb 24;14:1059. doi: 10.1038/s41467-023-36674-y (PMC9958100; doi:10.1038/s41467-023-36674-y)
Supplement: Supplementary file 1 — Supplementary Information [file 41467_2023_36674_MOESM1_ESM.pdf]

# SUPPLEMENTARY FIGURES

## A THOUSAND-GENOME PANEL RETRACES THE GLOBAL SPREAD AND ADAPTATION OF A MAJOR FUNGAL CROP PATHOGEN

Alice Feurtey et al.

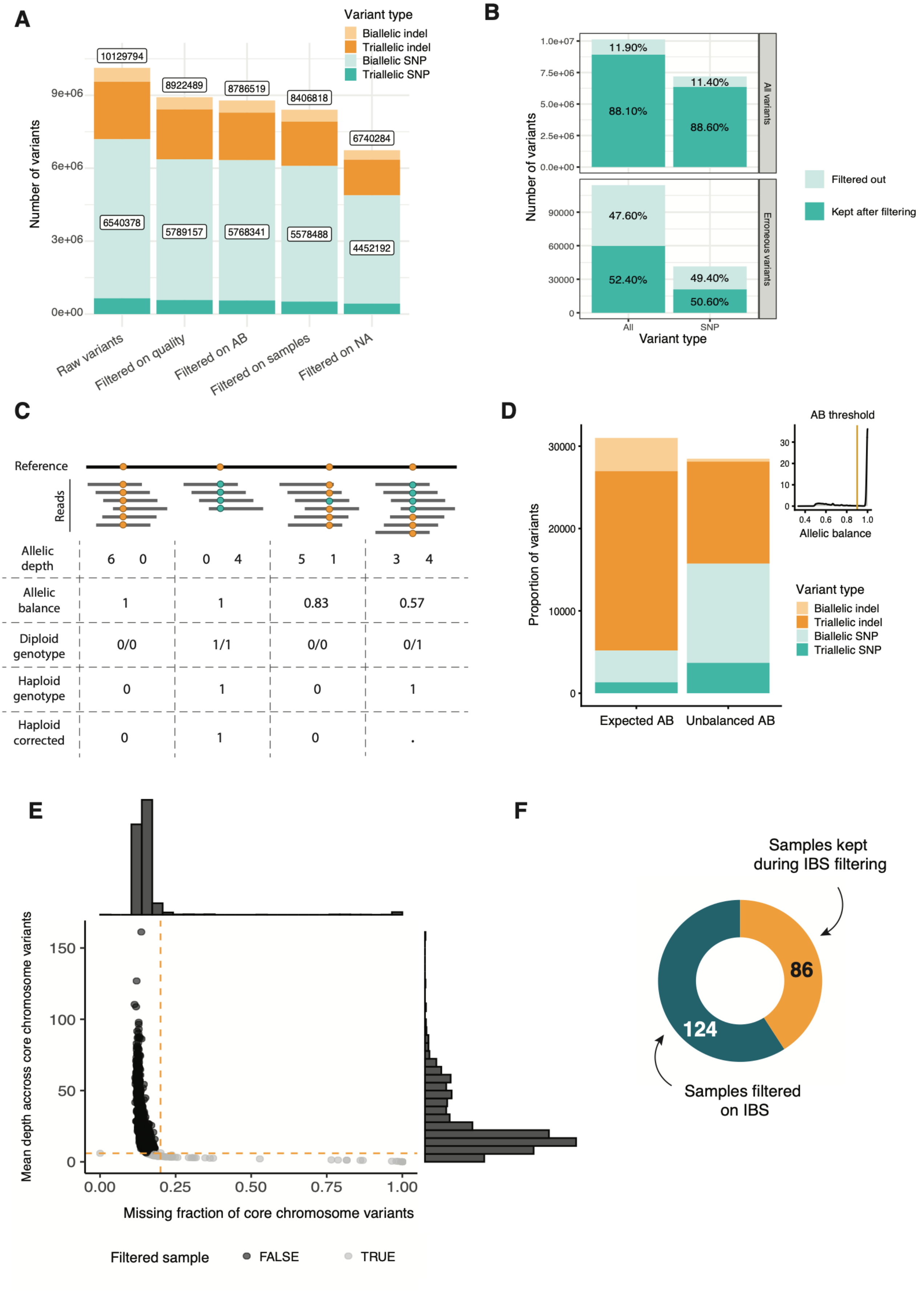


**Figure S1: Short variants filtering procedure.**

A. Number of short variants retained after each filtering step from the raw variant calling of GATK to the final filtered dataset.
B. Number and proportions of variants filtered by hard filtering on quality and depth per position. Top panel: all variants. Bottom panel: only erroneous variants detected through the analysis of replicate resequencing sets of the same isolate.
C. Schematic representation of the allelic balance (AB) and the associated filtering. The schematic shows a threshold of 0.8 as an example (we used 0.9 in our analyses). Four different scenarios of allelic depth, allelic balance, called diploid and haploid genotypes both pre- and post-filtering. The dot represents a genotype set to missing data during variant calling.
D. Number of variants of each type (colors as in panel A) for both variants found with a typical AB and with unbalanced AB, based on the threshold illustrated in the density plot of the upper right corner of the panel.
E. Filtering at the sample level based on mean depth of coverage and missing data proportions on core chromosomes.
F. Number of samples removed and kept amongst the 210 isolates with high relatedness to at least one other isolate.

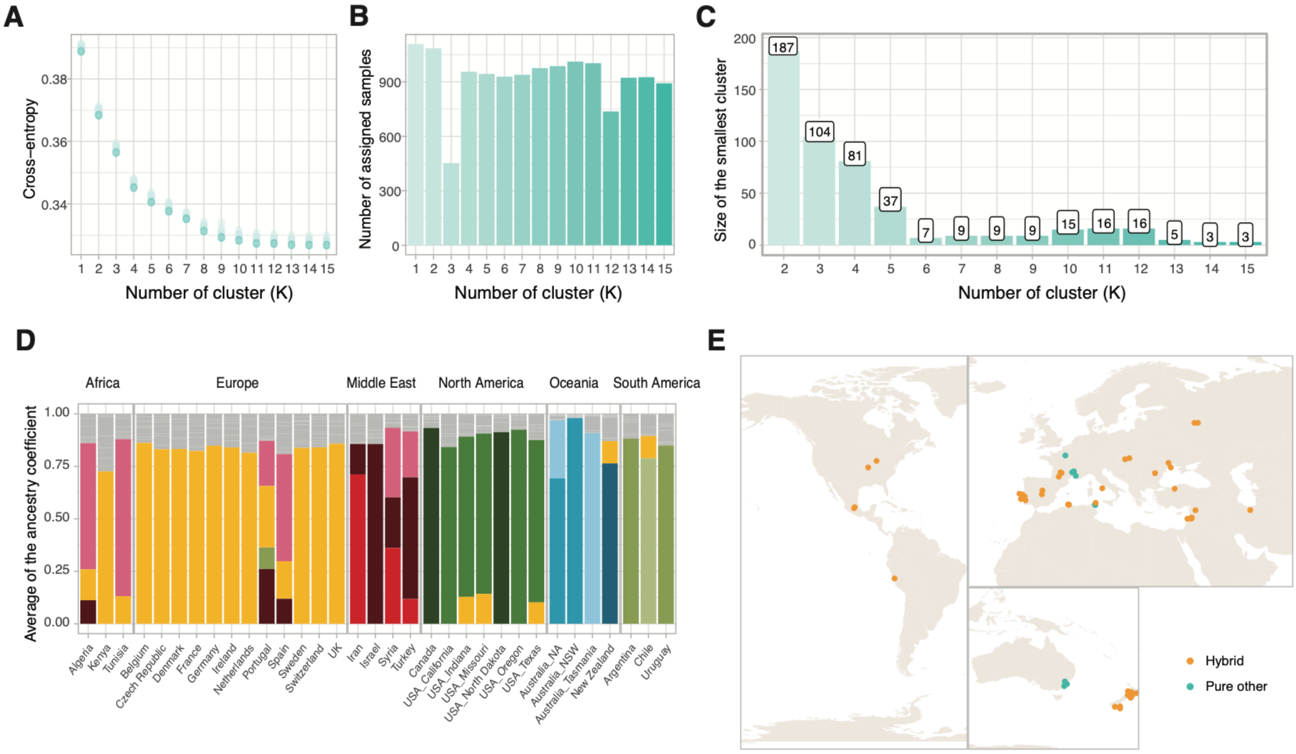


**Figure S2: Genetic clustering of 1109 *Zymoseptoria tritici* isolates and hybrid detection.**
A. Cross-entropy for each number of clusters (K) as assessed by the R package *LEA*.
B. Number of isolates uniquely assigned to one cluster per K.
C. Number of isolates assigned to the smallest cluster.
D. Barplots for the best K (as in panels A-C) per country and state (USA). Color represents the main clusters and correspond to the clusters shown in Figure 1.
E. Map representing the isolates identified either as hybrid or as fully belonging to a cluster that is not the typical local cluster for the country.

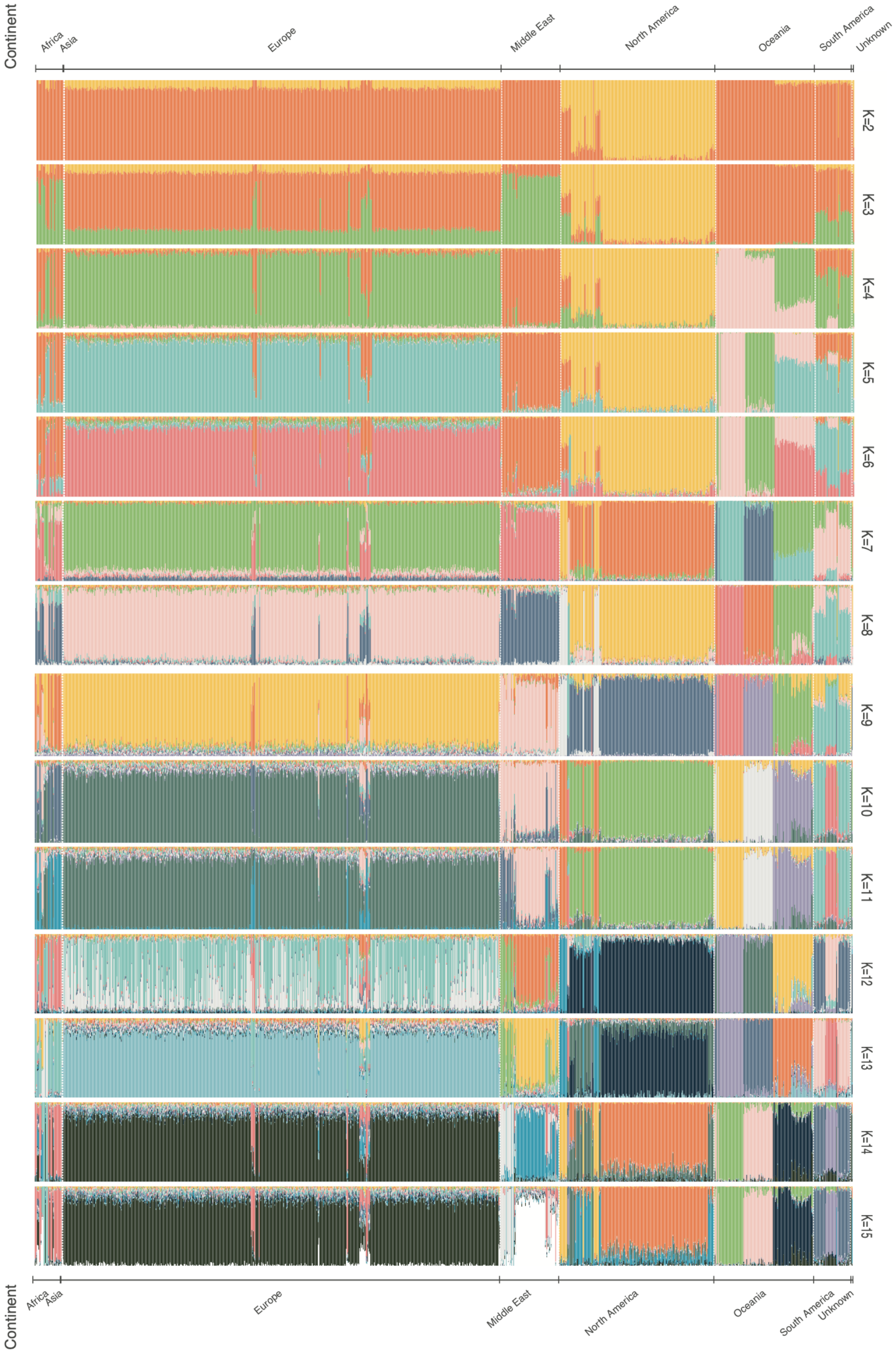


**Figure S3: Genetic clustering of 1109 *Zymoseptoria tritici* isolates showing per-isolate barplots with cluster assignments.** Barplots for the best replicate for each K (as determined by cross-entropy). Barplots are shown for each analyzed value of K. Each vertical bar represents an isolate and the clusters are represented by different colors. Isolates are sorted by continent and per country.

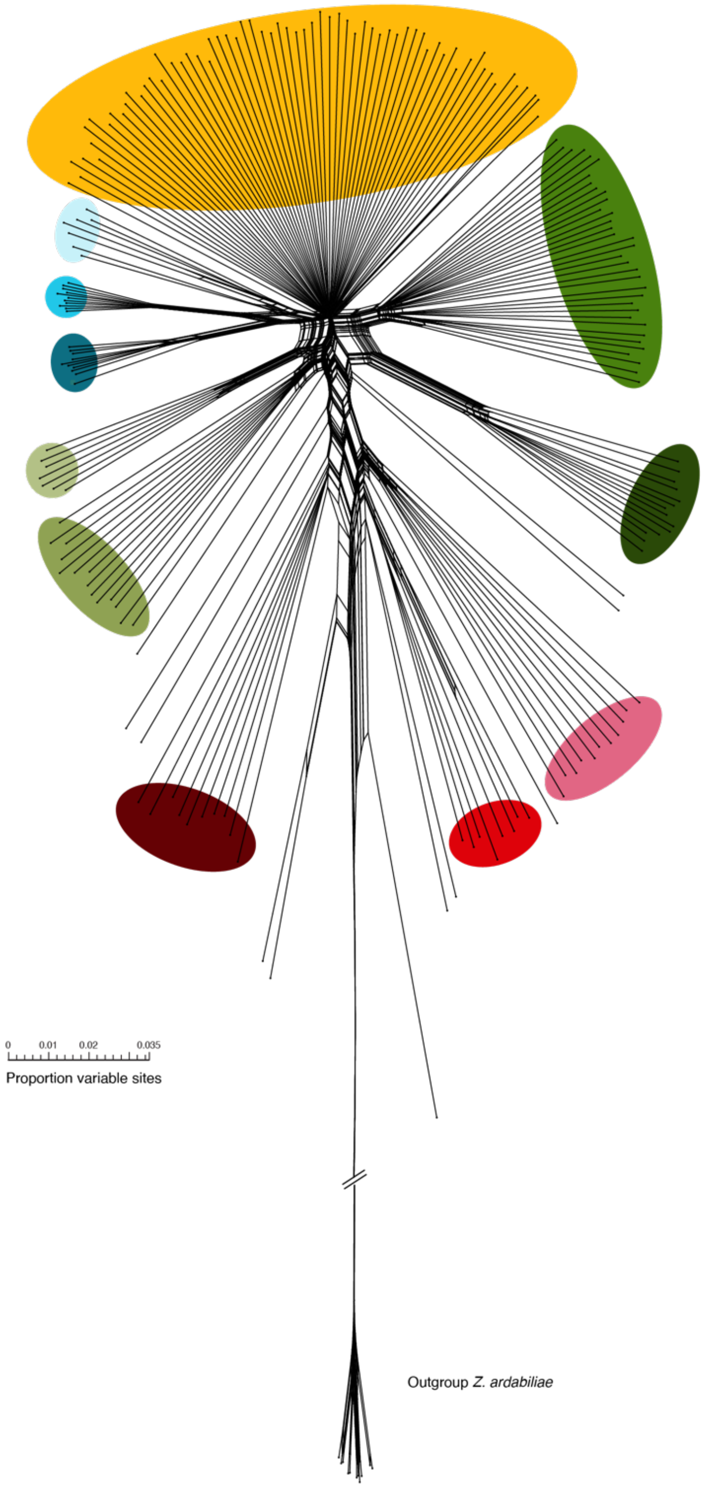


**Figure S4: SplitsTree phylogenetic network of a geographically representative subset of *Zymoseptoria tritici* isolates and *Z. ardabiliae* as an outgroup.** Circle colors correspond to cluster colors shown in Figure 2.

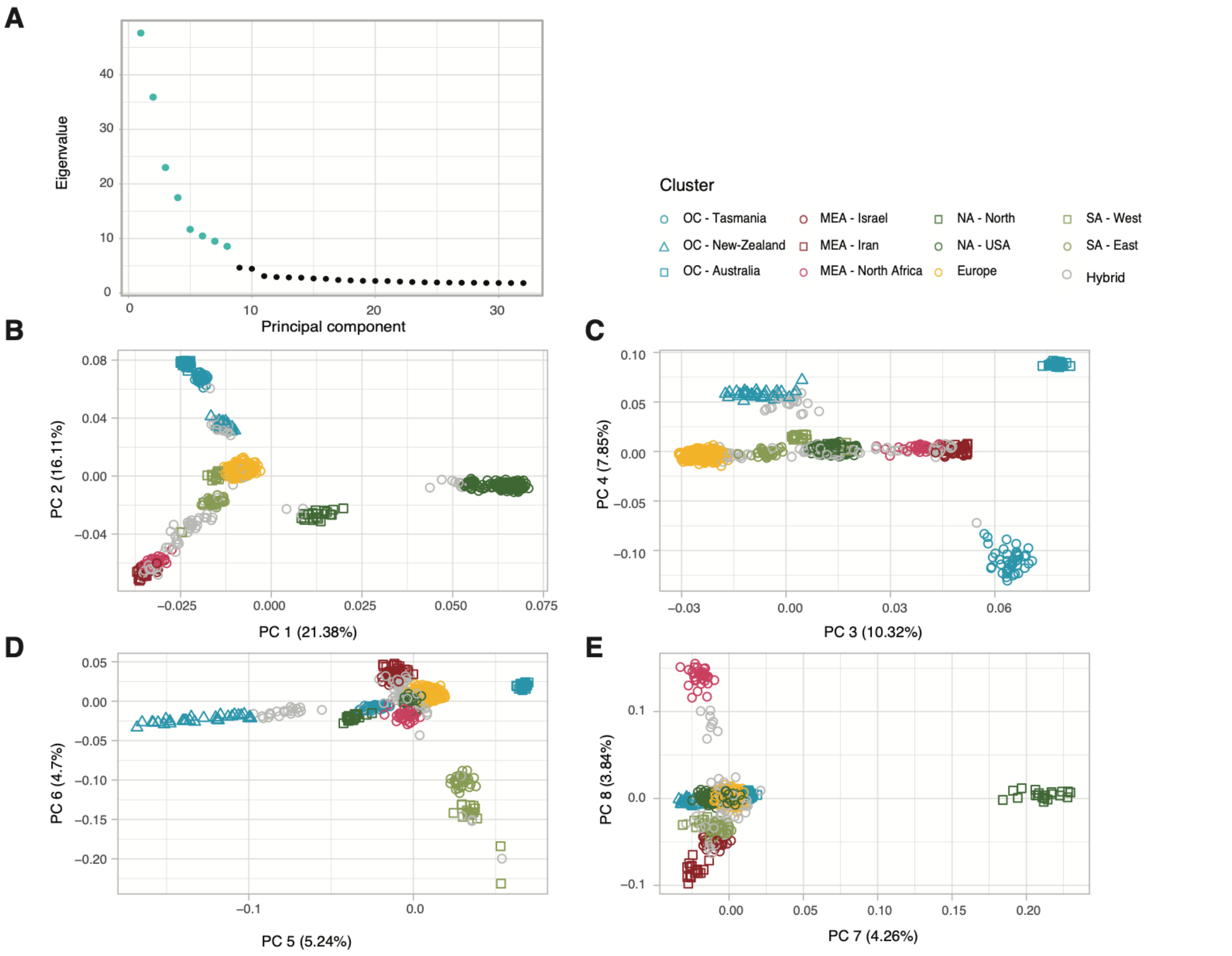


**Figure S5: Principal component analysis based on a subset of the short variant dataset.**

A. Eigenvalues per principal component (PC). The PCs visualized in panels B-E are shown in blue.

B - E. Pairs of PC1-8 with colors and shapes identifying the 11 clusters as defined by the clustering analysis. Genotypes shown in grey could not be attributed fully to any cluster and are thus classified as inter-cluster hybrids. Names of the clusters include an abbreviation of continents and a more precise geographical location (MEA: Middle-East and Africa; NA: North America; SA: South America; OC: Oceania).

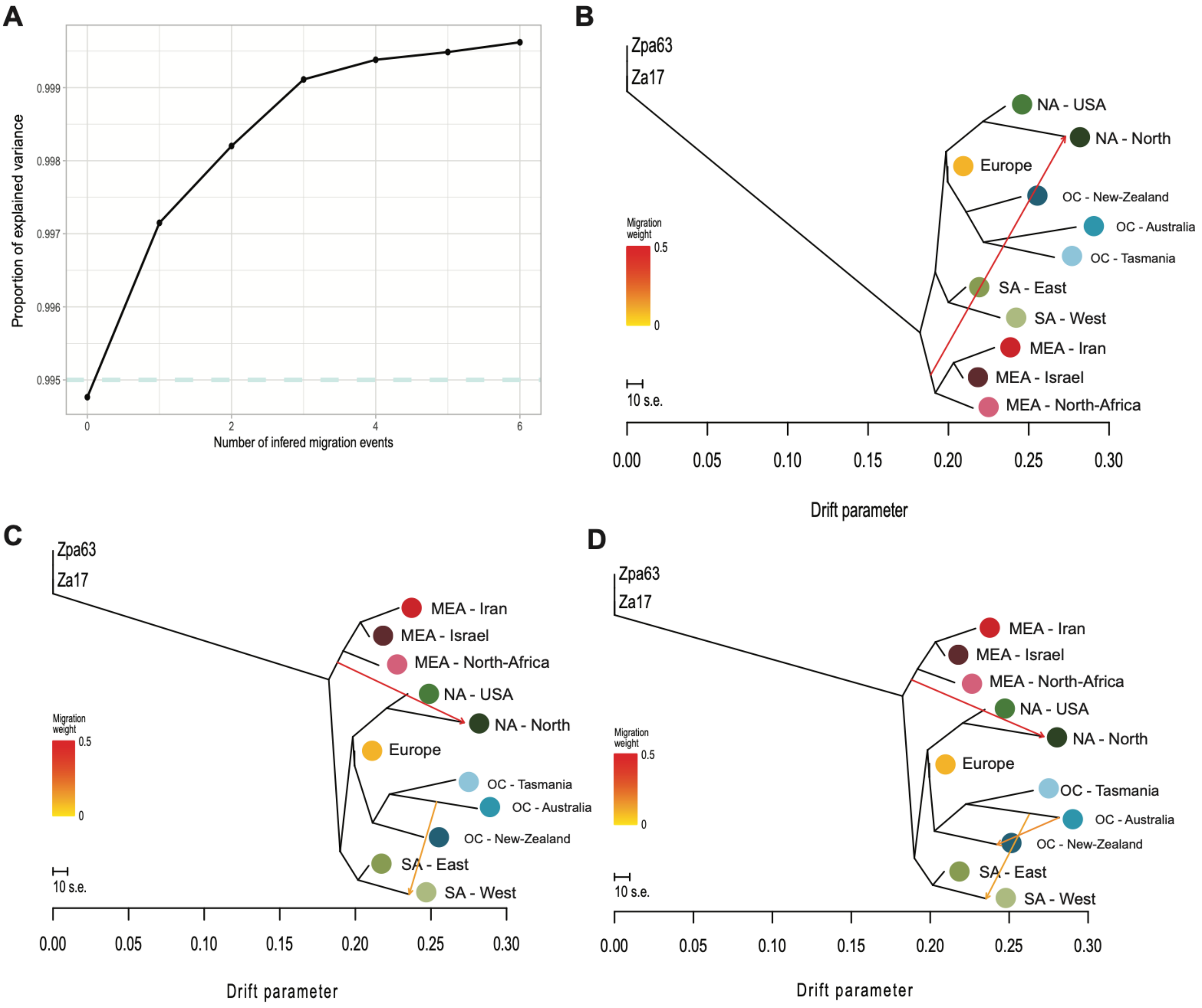


**Figure S6: Population trees (Treemix analysis) with various numbers of inferred migration events**

A. Proportion of explained variance assuming between 0-6 inferred migration events.
B. Tree representation for 1 migration event. Colors of the dot are identical to the colors of the cluster in figure 2 (and other figures). Names of the clusters include an abbreviation of continents and a more precise geographical location (MEA: Middle-East and Africa; NA: North America; SA: South America; OC: Oceania).
C. Tree representation for 2 migration events.
D. Tree representation for 3 migration events.

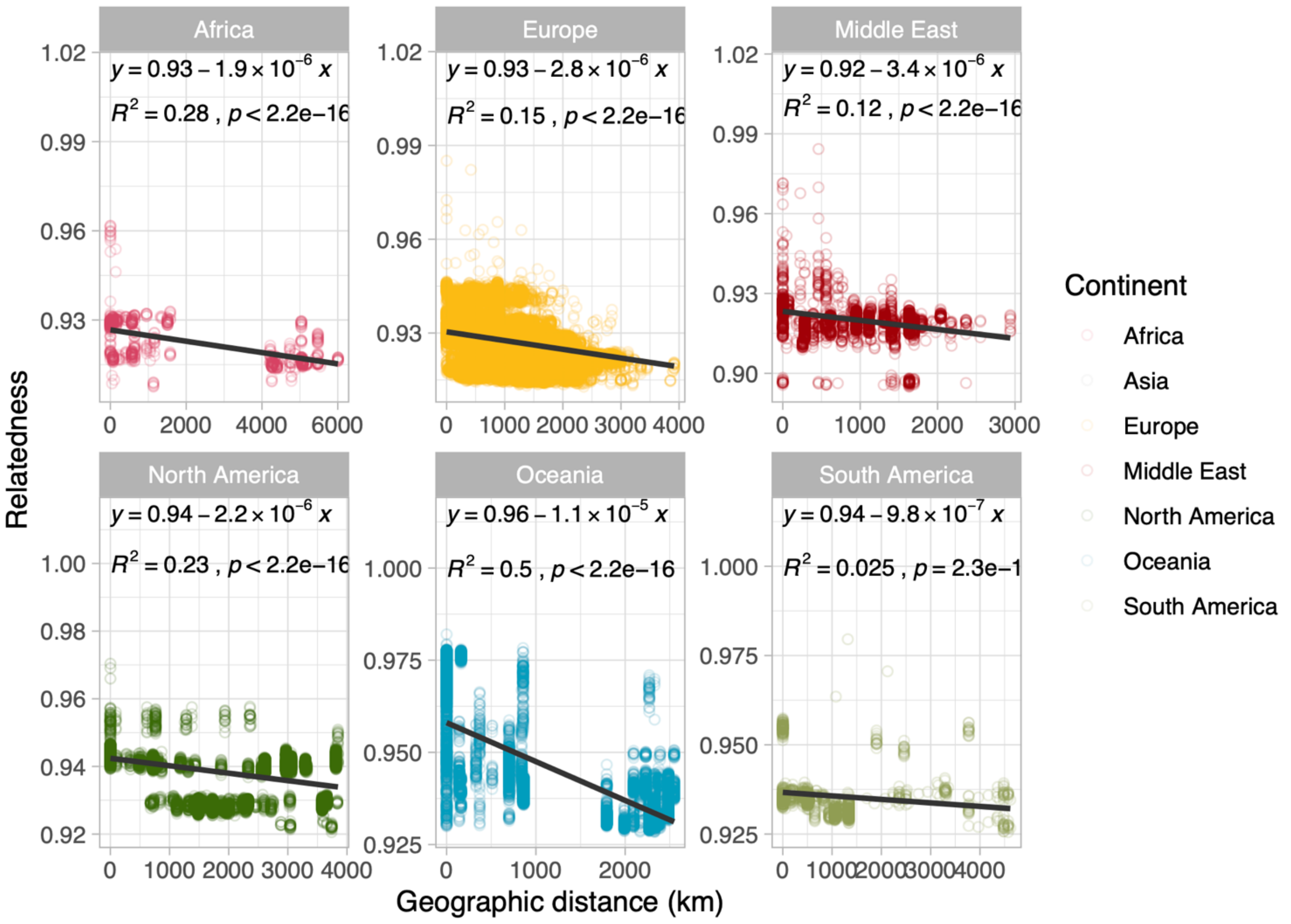


**Figure S7: Isolation-by-distance per continent assessed by Pearson correlations between relatedness and geographic distance between sampling locations**. Each dot represents an isolate.

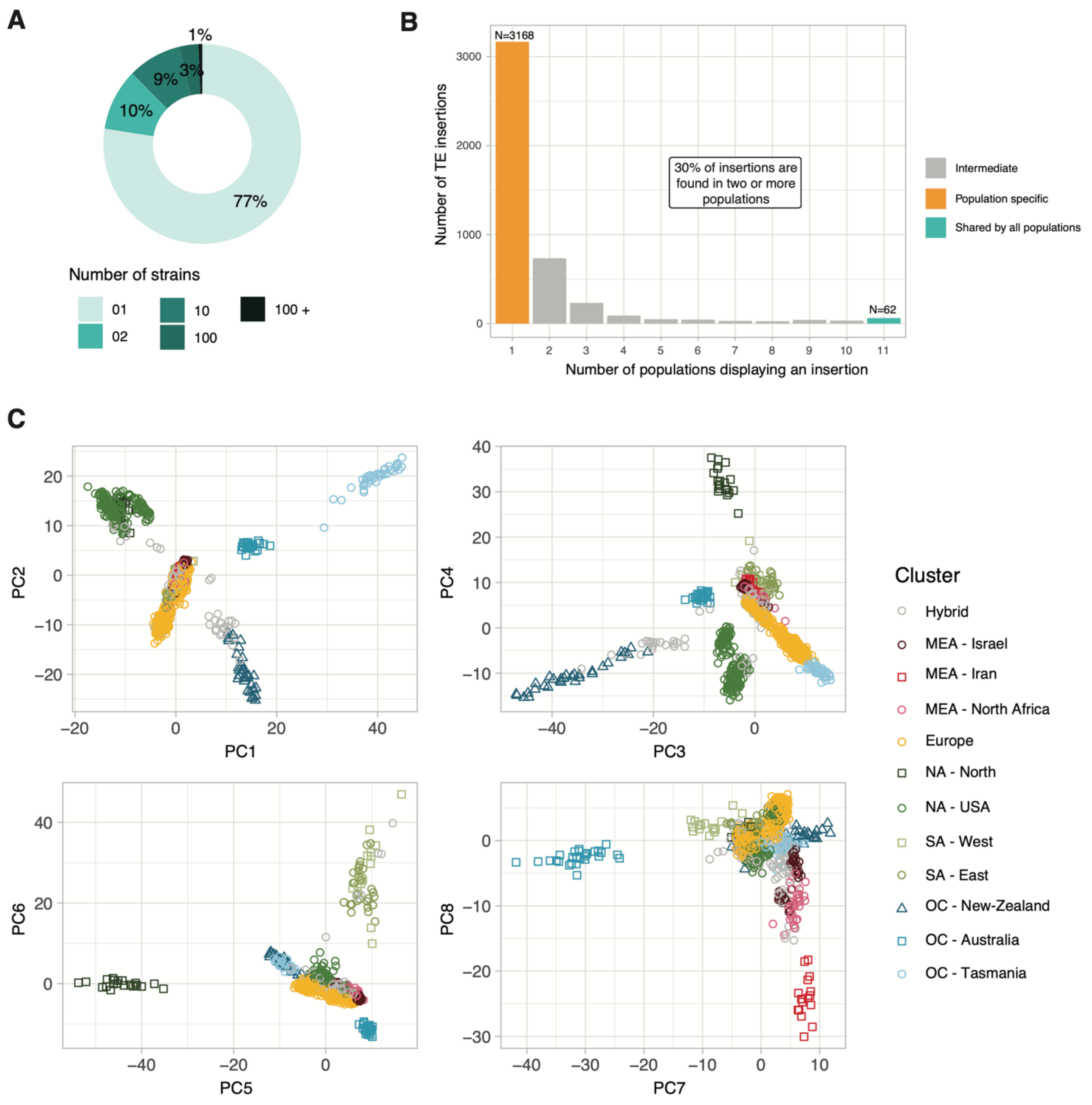


**Figure S8: Transposable element insertion polymorphisms (TIPs).**

A. Proportion of TIPs shared by different numbers of isolates.
B. Number of TIPs found uniquely in one population (orange), found in a subset of populations (grey) or all populations (green).
C. Principal component analysis (PC 1 to 8) based on TIPs shared by at least 10 isolates. Each point represents an isolate. The shape and colors of the dots represent the genetic clusters identified based on the short variant polymorphisms. Names of the clusters include an abbreviation of continents and a more precise geographical location (MEA: Middle-East and Africa; NA: North America; SA: South America; OC: Oceania).

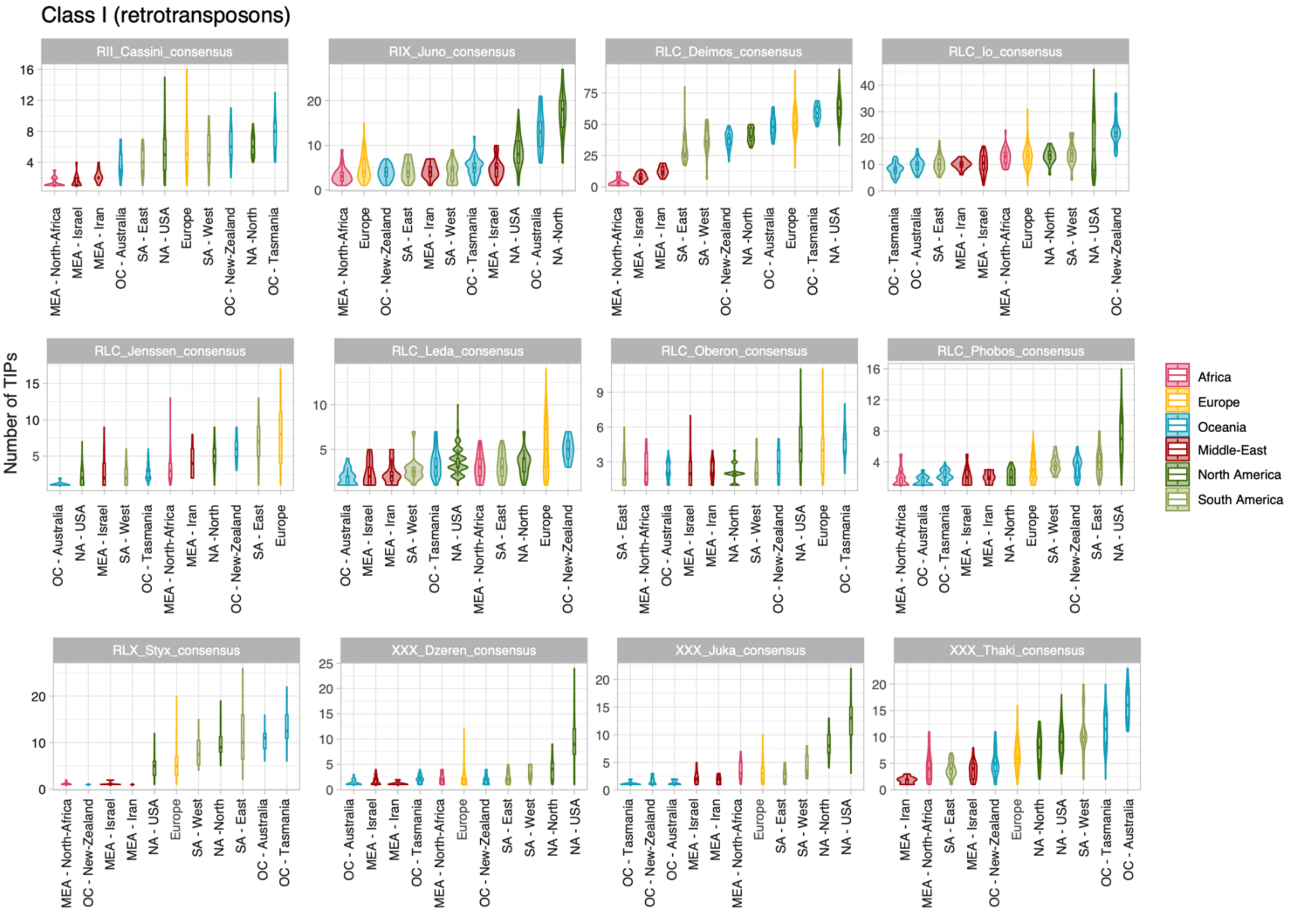


**Figure S9: Number of transposable element insertion polymorphisms (TIPs) for class I retrotransposons per cluster.** Only elements with at least 10 TIPs overall are shown (*n* = 977 isolates). The lower and upper hinges of the boxplots correspond to the first and third quartiles, the whiskers to the largest value are within 1.5 times the inter-quartile range and the central horizontal line defines the median.

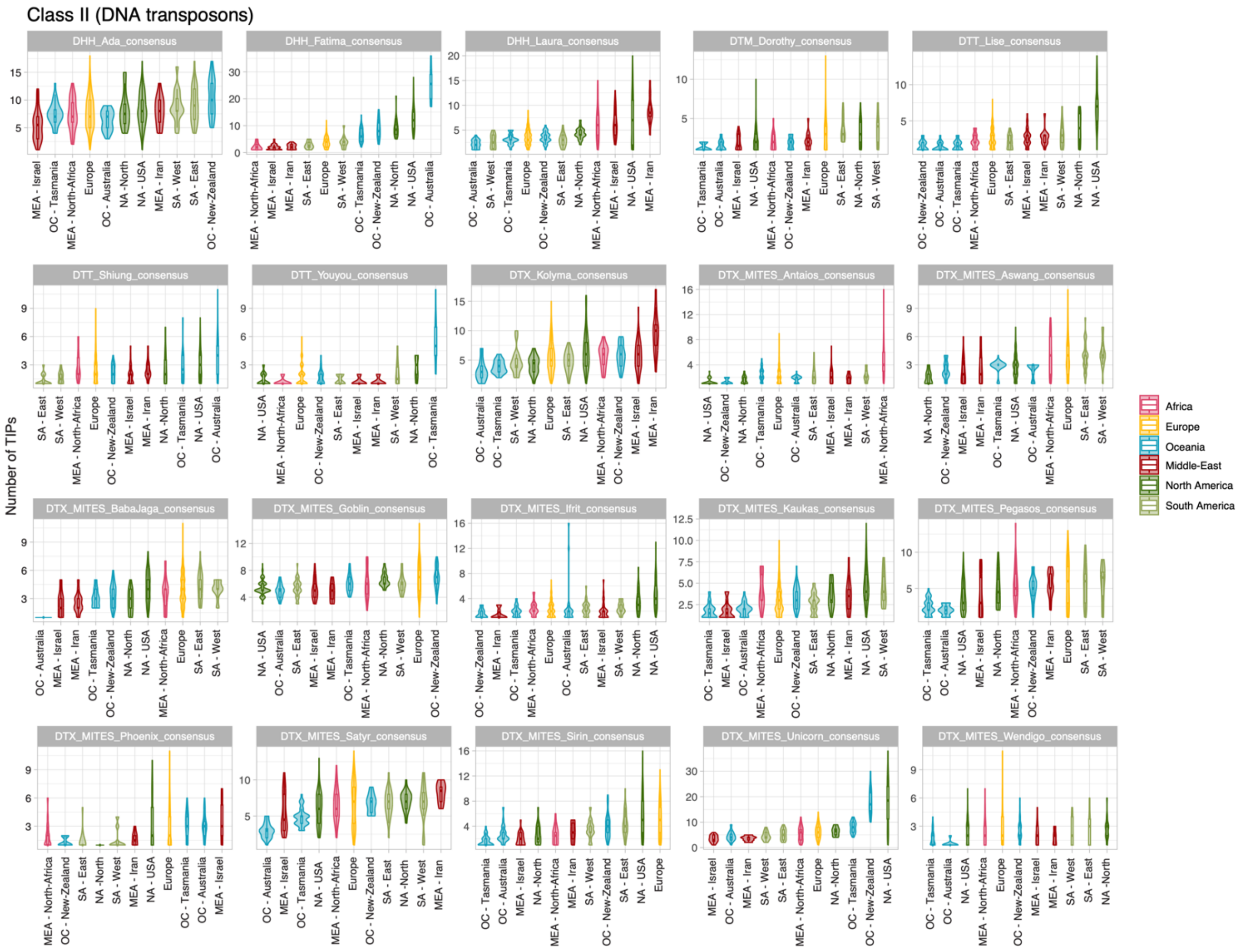


**Figure S10: Number of transposable element insertion polymorphisms (TIPs) for class II transposons per cluster.** Only elements with at least 10 TIPs overall are shown ($n$ = 977 isolates). The lower and upper hinges of the boxplots correspond to the first and third quartiles, the whiskers to the largest value are within 1.5 times the inter-quartile range and the central horizontal line defines the median.

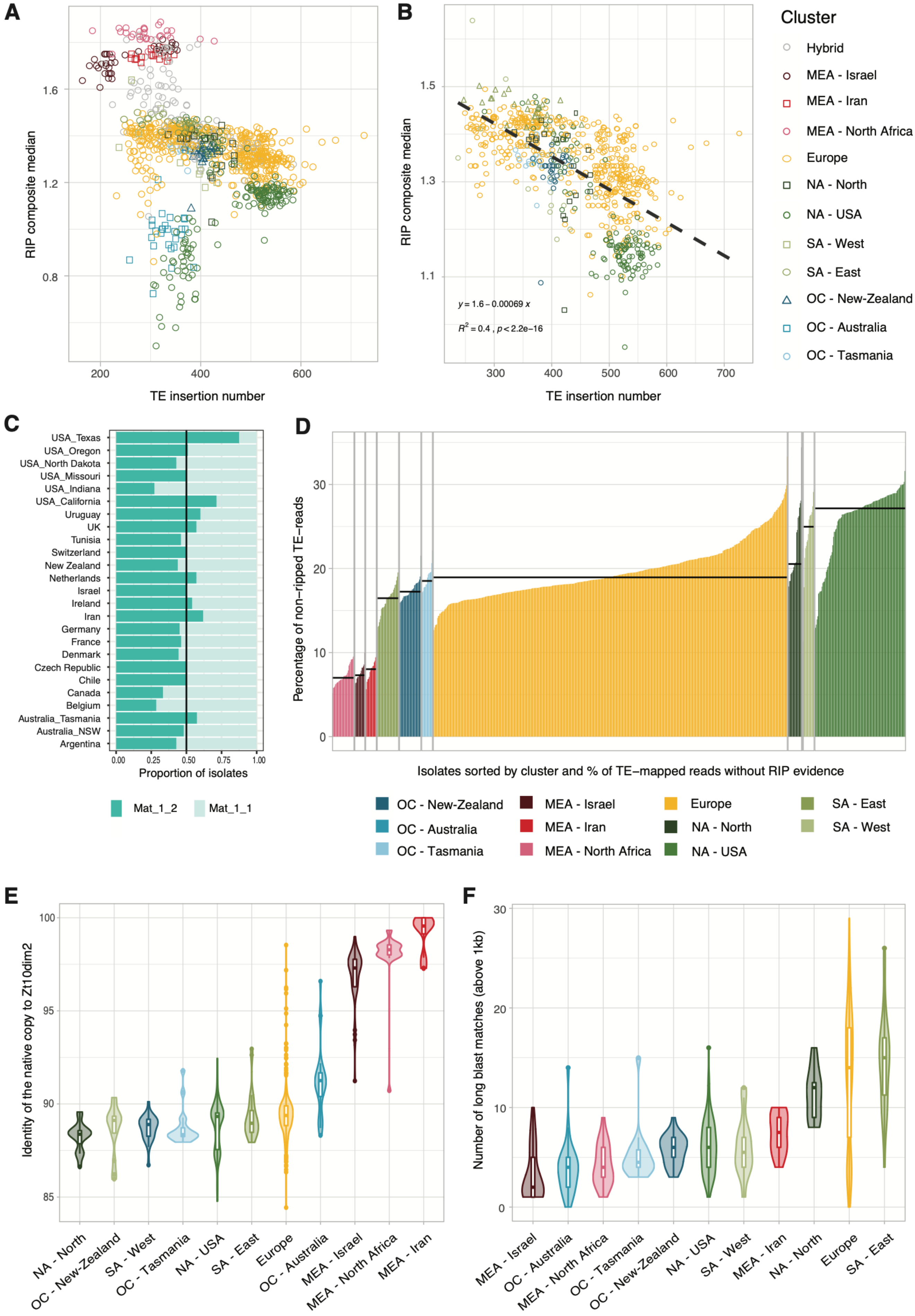

A
B
RIP composite median
TE insertion number
y = 1.6 − 0.00069 x
R² = 0.4 , p < 2.2e−16
Cluster
Hybrid
MEA - Israel
MEA - Iran
MEA - North Africa
Europe
NA - North
NA - USA
SA - West
SA - East
OC - New-Zealand
OC - Australia
OC - Tasmania
C
USA_Texas
USA_Oregon
USA_North Dakota
USA_Missouri
USA_Indiana
USA_California
Uruguay
UK
Tunisia
Switzerland
New Zealand
Netherlands
Israel
Ireland
Iran
Germany
France
Denmark
Czech Republic
Chile
Canada
Belgium
Australia_Tasmania
Australia_NSW
Argentina
Proportion of isolates
Mat_1_2
Mat_1_1
D
Percentage of non−ripped TE−reads
Isolates sorted by cluster and % of TE−mapped reads without RIP evidence
E
Identity of the native copy to Zt10dim2
F
Number of long blast matches (above 1kb)

**Figure S11: Patterns of repeat-induced point mutations (RIP) across populations**

A. Mean RIP composite index in TE-mapped reads and TE insertion counts in the global collection of genomes. Each dot represents a different isolate. Colors and shape of dots in panels A and B correspond to the genetic clusters identified based on short variant polymorphisms. Names of the clusters include an abbreviation of continents and a more precise geographical location (MEA: Middle-East and Africa; NA: North America; SA: South America; OC: Oceania).
B. Correlation between the RIP composite index in TE-mapped reads and TE insertion counts per genome (excluding the Middle East/Africa clusters; Pearson correlation coefficient).
C. Proportions of isolates of different mating types (carrying either Mat_1_2 and Mat_1_1) per country/state. The black line represents balanced proportions among mating types (*i.e.* 0.5).
D. Percentage of TE-mapped reads per isolate showing no evidence of RIP (based on a RIP composite index threshold of 0.5). The median percentage per genetic cluster is represented as a horizontal black line.
E. Violin plots and boxplots representing the identity of the native copy of *dim2* per cluster in 902 draft genome assemblies of individual isolates in which *dim2* was identified (based on a BLASTn search and using the flanking genes to identify the native locus). The lower and upper hinges of the boxplots correspond to the first and third quartiles, the whiskers to the largest value are within 1.5 times the inter-quartile range and the central horizontal line defines the median.
F. Violin plots and boxplots representing the identity (%) and number of long BLASTn matches (>1 kb) of the functional copy of *dim2* in 902 draft genome assemblies of individual isolates grouped by genetic cluster. The lower and upper hinges of the boxplots correspond to the first and third quartiles, the whiskers to the largest value are within 1.5 times the inter-quartile range and the central horizontal line defines the median.

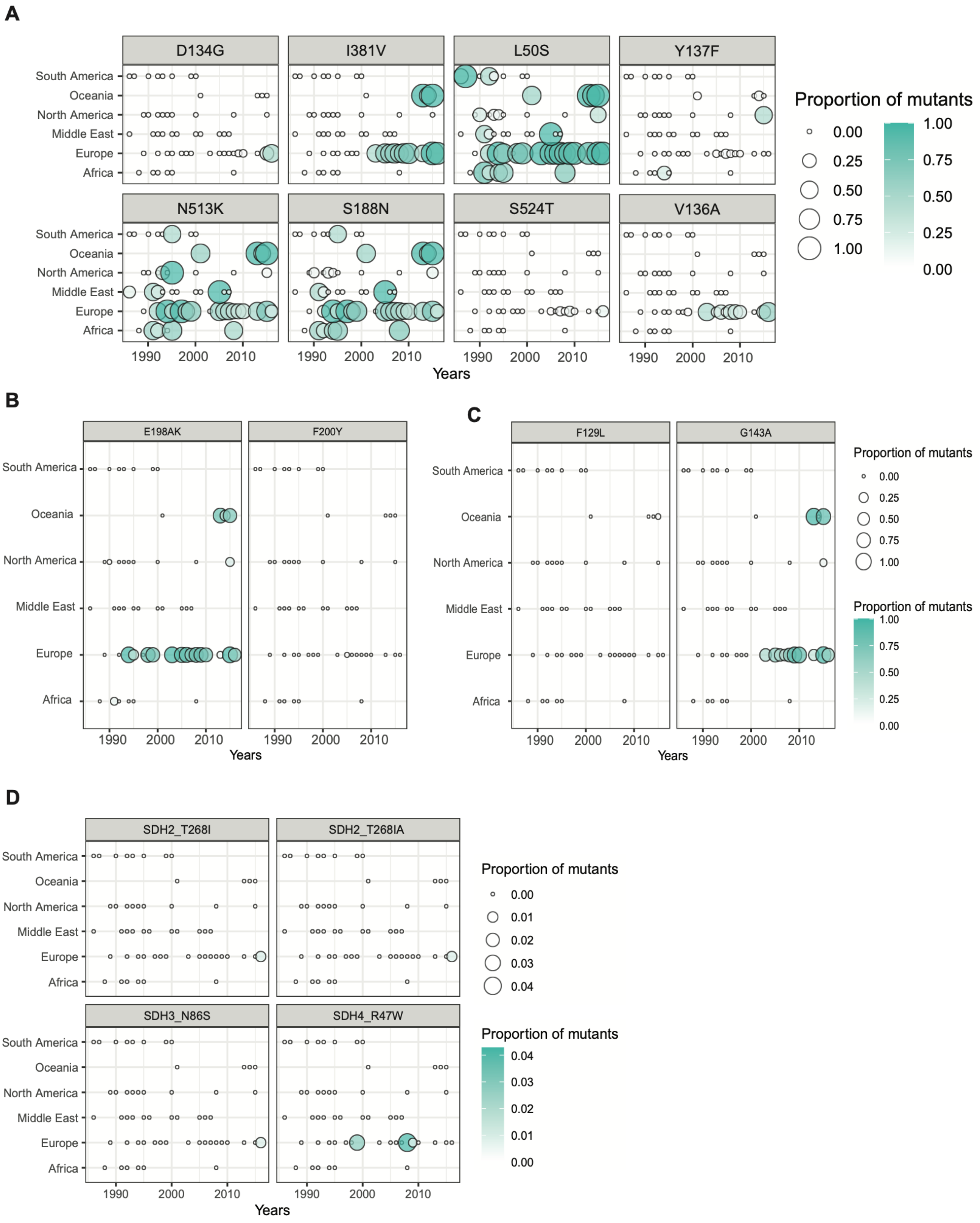


**Figure S12: Survey of mutations known to be associated with resistance to fungicides or observed in natural populations in gene known to be involved in resistance**

A. Mutations in the *cyp51* gene, known to be involved in azole resistance.
B. Mutations in the beta tubulin gene, known to be involved in resistance to benzimidazole fungicides.
C. Mutations in the mitochondrial gene *cytb*, known to cause resistance to Quinone outside inhibitors fungicides.
D. Mutations in the SDH genes, known to be involved in resistance to SDHI fungicides.

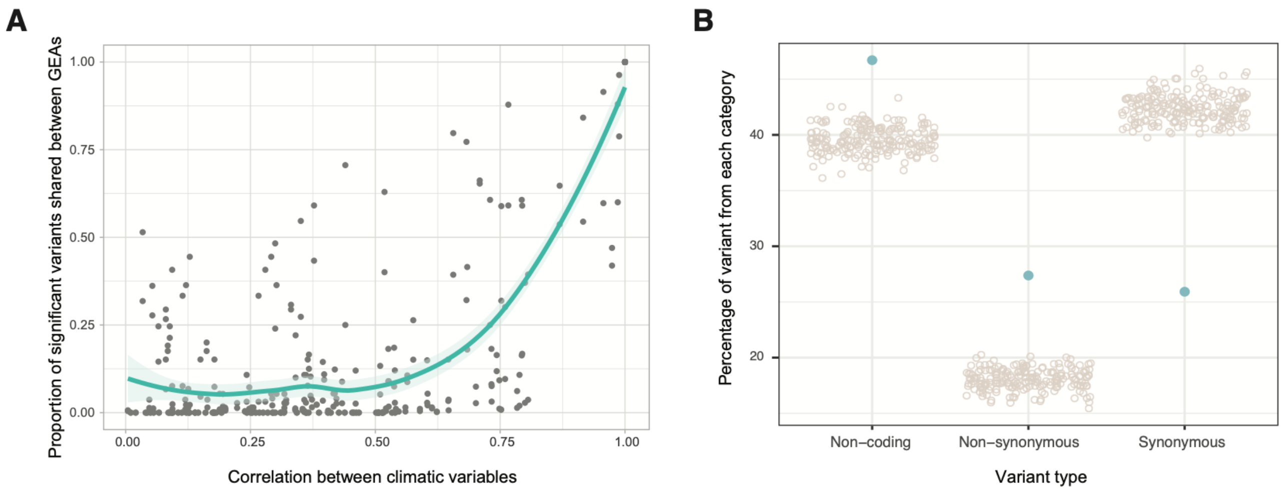


**Figure S13: Genome-environment association mapping: similarity between environmental variables and impact of the significantly associated variants on the predicted protein sequences.**

A. Dot plot showing the correlation between bioclimatic variables and the proportion of variants detected as significantly associated to two bioclimatic variables. The green curve was obtained with the loess method, which fits a polynomial using local fitting, span = .75. The lighter green band shows the confidence interval with the default value (0.95).
B. Comparison between the percentage of variants in non-coding regions or having synonymous or non-synonymous effects (in green) compared against 200 randomly sampled short variants (with a MAF > 0.01 to match the threshold used for the GEA).

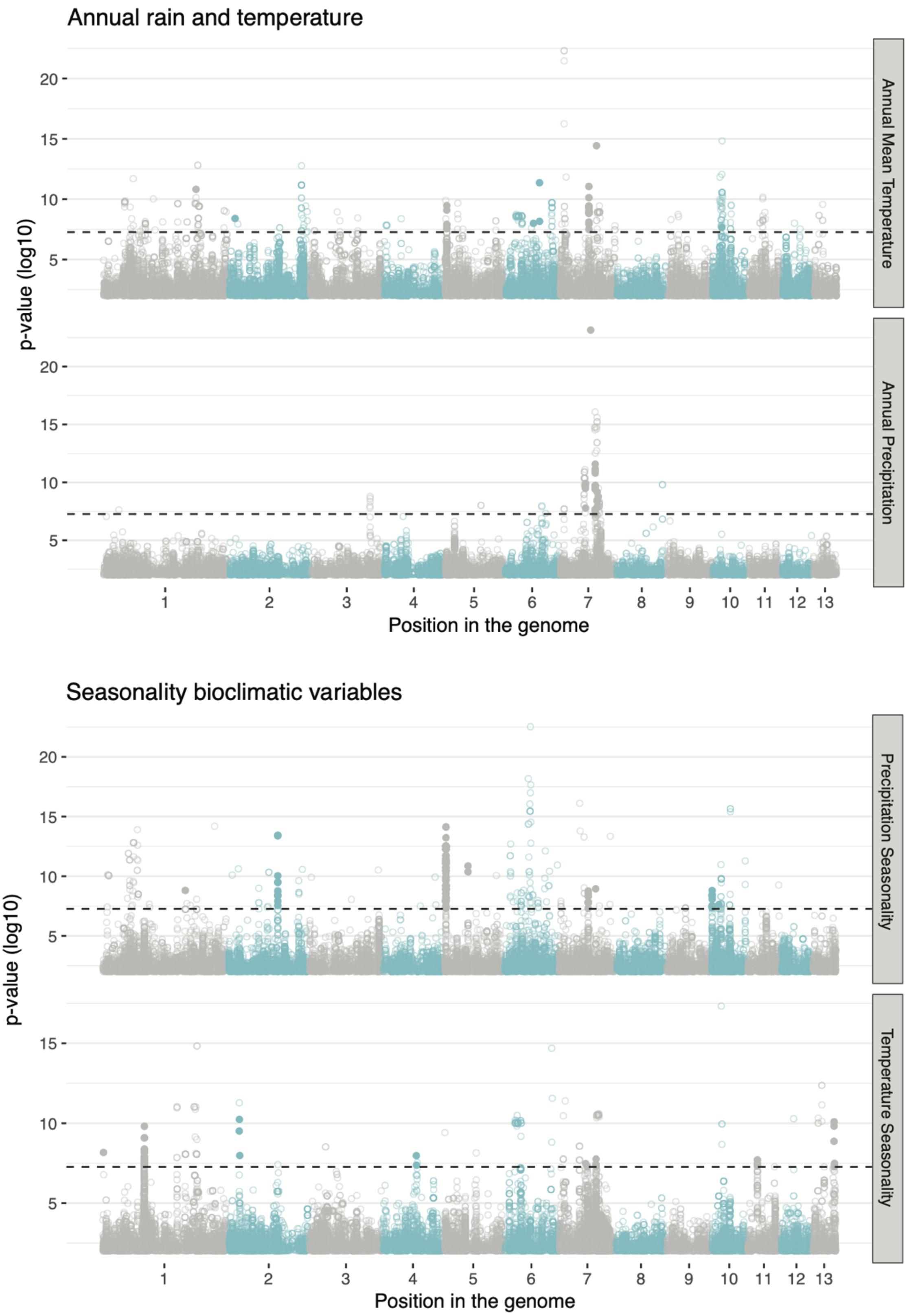


**Figures S14: Manhattan plots of the GEA for bioclimatic variables related to annual rain and temperature levels and to seasonality.** The x-axis represents the genomic position, each dot represents a short variant with the colors showing alternating chromosomes. Values obtained from the software GEMMA with the default linear model (Wald test). The black dashed line is the Bonferroni threshold. Each variant above the threshold is either a filled circle to show a minor allele frequency (MAF) above 5% or an empty circle for lower MAF.

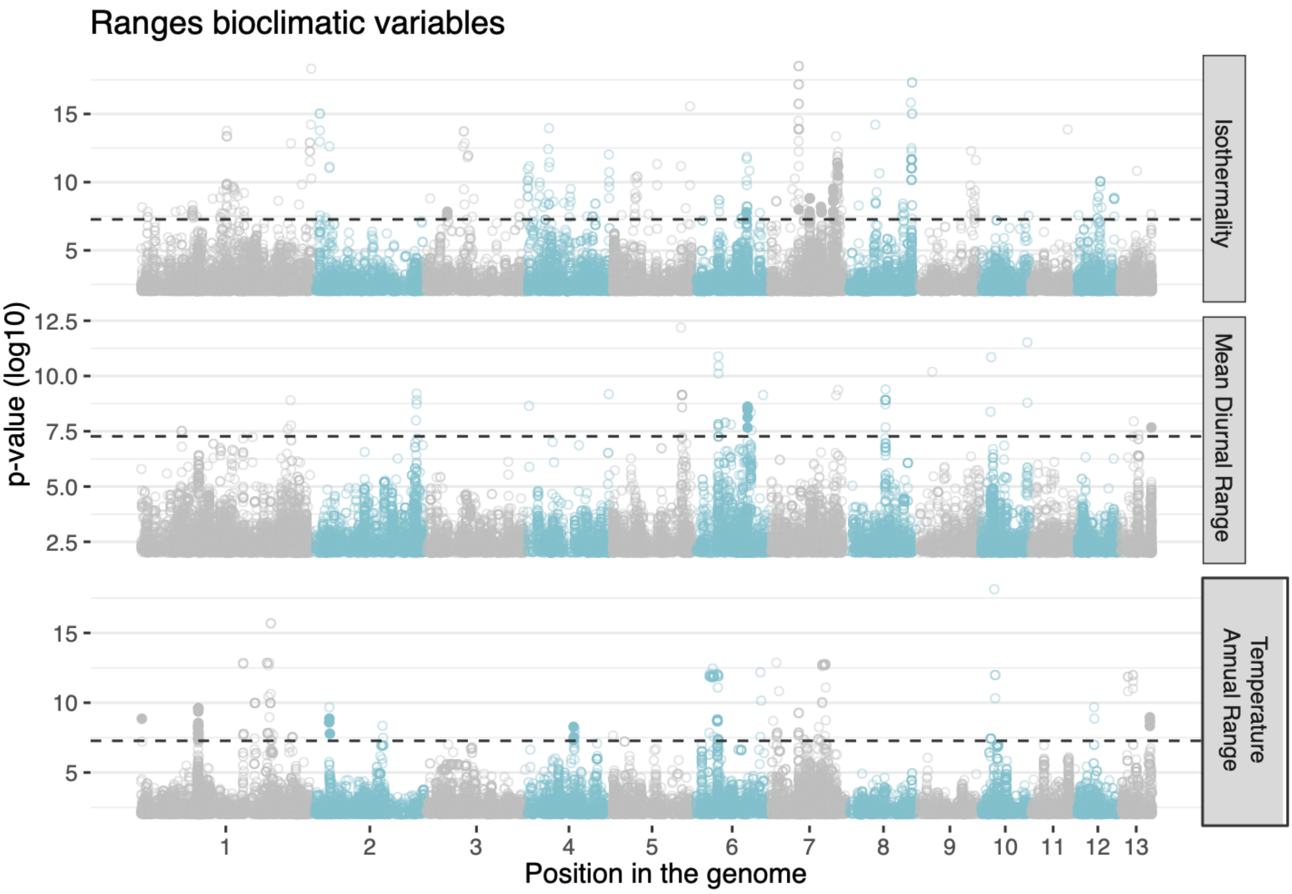


**Figures S15: Manhattan plots of the GEA for the iso-thermality, the mean diurnal range and the annual temperature range.** The x-axis represents the genomic position, each dot represents a short variant with the colors showing alternating chromosomes. Values obtained from the software GEMMA with the default linear model (Wald test). The black dashed line is the Bonferroni threshold. Each variant above the threshold is either a filled circle to show a minor allele frequency (MAF) above 5% or an empty circle for lower MAF.

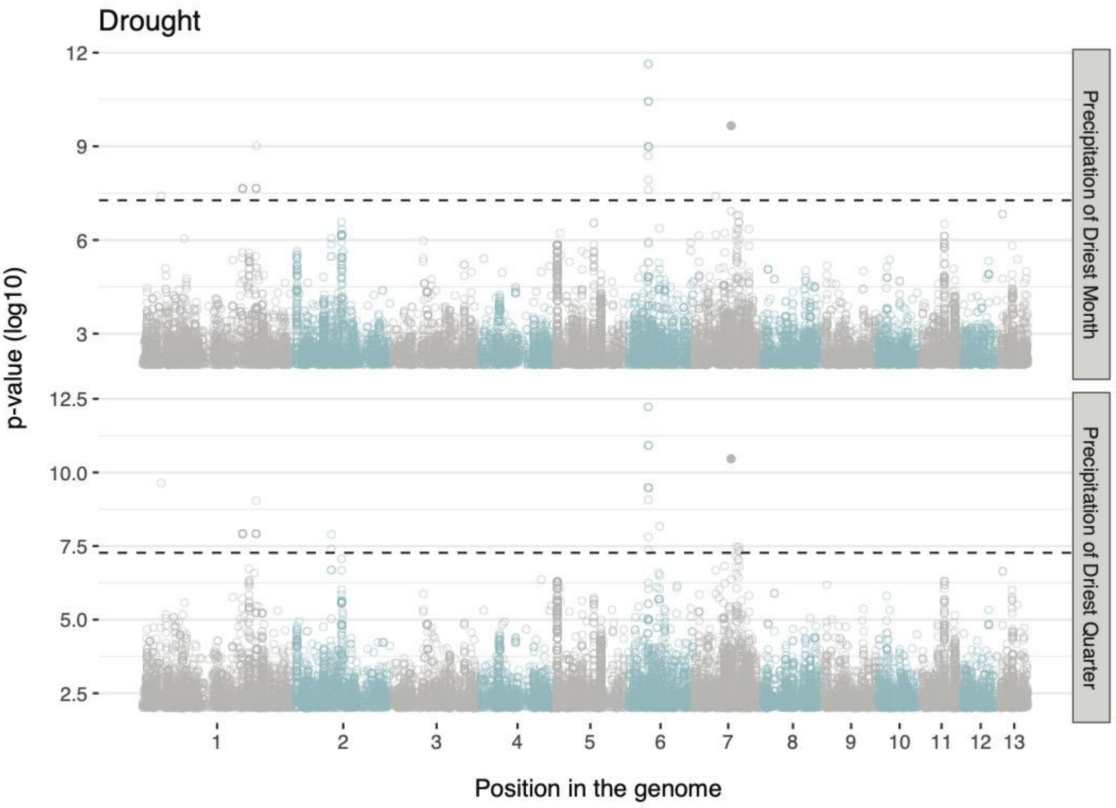


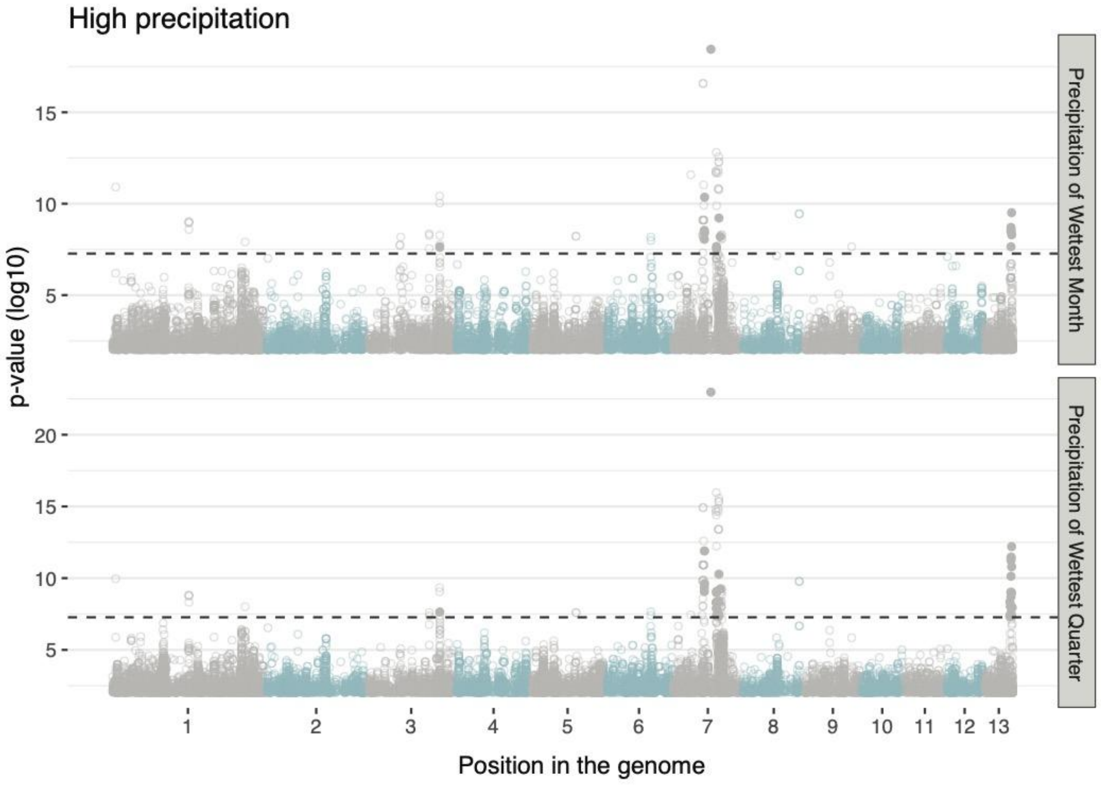


**Figures S16: Manhattan plots of the GEA for high and low precipitation variables.**
The x-axis represents the genomic position, each dot represents a short variant with the colors showing alternating chromosomes. Values obtained from the software GEMMA with the default linear model (Wald test). The black dashed line is the Bonferroni threshold. Each variant above the threshold is either a filled circle to show a minor allele frequency (MAF) above 5% or an empty circle for lower MAF.

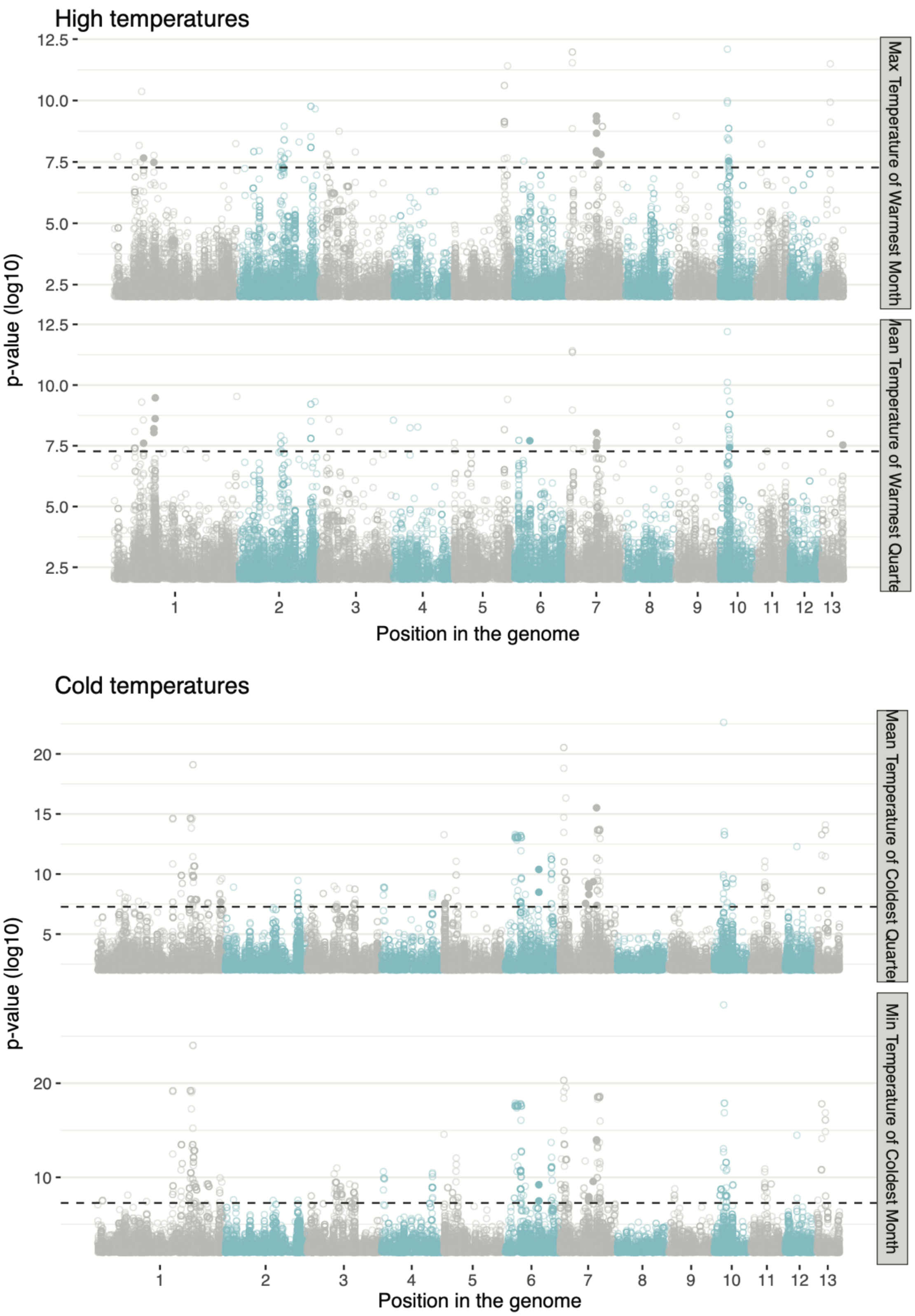


**Figures S17: Manhattan plots of the GEA for high and low temperature variables.**
The x-axis represents the genomic position, each dot represents a short variant with the colors showing alternating chromosomes. Values obtained from the software GEMMA with the default linear model (Wald test). The black dashed line is the Bonferroni threshold. Each variant above the threshold is either a filled circle to show a minor allele frequency (MAF) above 5% or an empty circle for lower MAF.

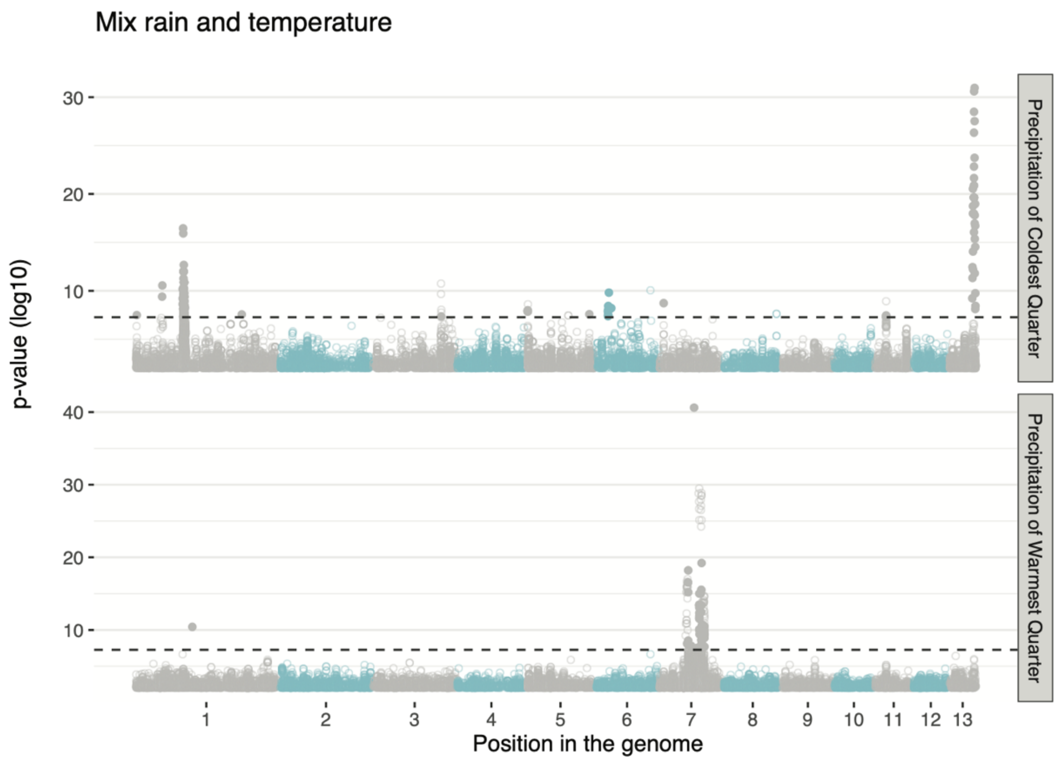


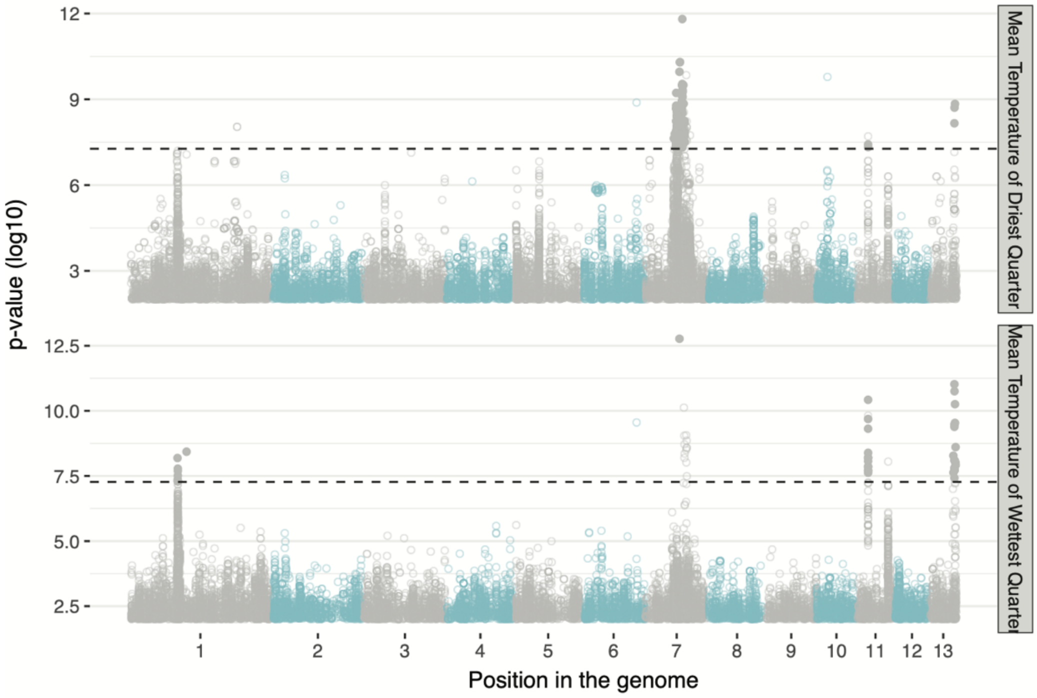


**Figures S18: Manhattan plots of the GEA for bioclimatic variables that include information about both precipitation and temperature.** The x-axis represents the genomic position, each dot represents a short variant with the colors showing alternating chromosomes. Values obtained from the software GEMMA with the default linear model (Wald test). The black dashed line is the Bonferroni threshold. Each variant above the threshold is either a filled circle to show a minor allele frequency (MAF) above 5% or an empty circle for lower MAF.

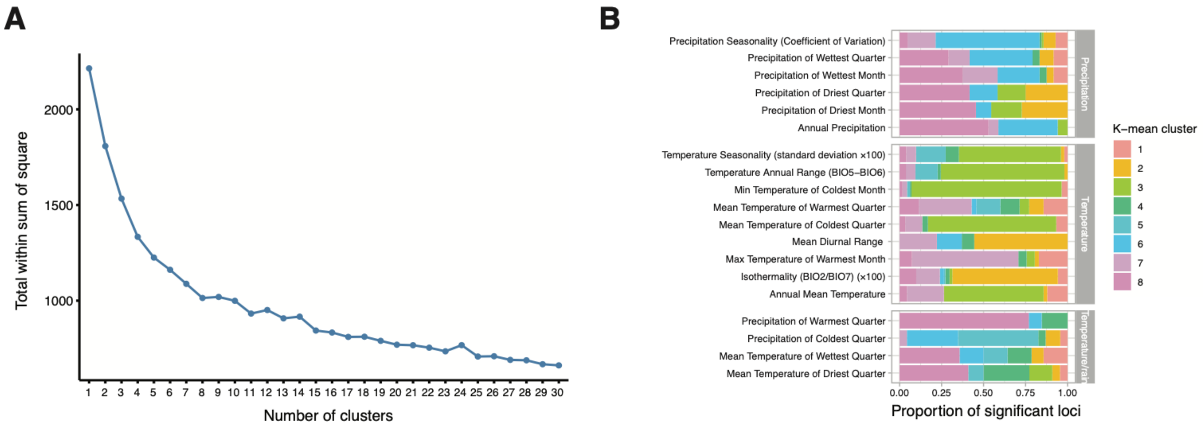


**Figure S19: K-mean clustering of the significant loci based on the presence-absence of the top allele in populations.**

A. Visualization of the total within cluster sum of square, which measures the compactness of the clustering, for cluster numbers ranging 1-30. Using the elbow method, we estimated the best number of clusters to be 8.
B. Proportion of variants belonging to either of the 8 clusters (colors) per bioclimatic variable.
