# Supplementary material for: A thousand-genome panel retraces the global spread and adaptation of a major fungal crop pathogen
Source: Nat Commun. 2023 Feb 24;14:1059. doi: 10.1038/s41467-023-36674-y (PMC9958100; doi:10.1038/s41467-023-36674-y)
Supplement: Supplementary file 3 — Description of Additional Supplementary Files [file 41467_2023_36674_MOESM3_ESM.pdf]

Description of Additional Supplementary Files

File Name: Supplementary Data 1
Description: Isolate metadata, including the isolate name, possible alternative names used in other publications, geographical location of the sampling site and inferred coordinates, sampling year, filtering status (kept/excluded for the analyses in the manuscript) as well as the NCBI Bioproject corresponding to the sequencing data.

File Name: Supplementary Data 2
Description: Sequencing depth per isolate and per chromosomes, as well as the median on the core chromosomes.

File Name: Supplementary Data 3
Description: Clustering results from the population structure. Each line reports the cluster membership value for each cluster and the cluster we assigned it to (based on a 0.75 threshold) or NA if the isolate was considered a hybrid/admixed genotype.

File Name: Supplementary Data 4
Description: Bioclimatic variables and their number of significantly associated variants and loci.

File Name: Supplementary Data 5
Description: Genes impacted by the significant variants from the GEA analysis.

File Name: Supplementary Data 6
Description: Overlap between the GEA significant loci and the QTL from Lendenmann et al.

File Name: Supplementary Data 7
Description: Summary statistics of the de novo draft assemblies obtained from Illumina resequencing datasets.
